# Supplementary material for: A Systematic Review of Circulating Tumor Cells Clinical Application in Prostate Cancer Diagnosis
Source: Cancers (Basel). 2022 Aug 4;14(15):3802. doi: 10.3390/cancers14153802 (PMC9367494; doi:10.3390/cancers14153802)
Supplement: Supplementary file 1 [file cancers-14-03802-s001.zip › cancers-1810791-supplementary.pdf]

Supplementary materials

Table S1. CTC in metastatic PC.

Author, year	Title	LE	Number of patients/samples	Conditions	Methodology	Cell count	Genes/receptors
T. Okegawa et al., 2016. [34]	Epidermal Growth Factor Receptor Status in Circulating Tumor Cells as a Predictive Biomarker of Sensitivity in Castration-Resistant Prostate Cancer Patients Treated with Docetaxel Chemotherapy	4	60 patients	mCRPC at docetaxel. Mean OS 13.7 ± 9.2 mo.	CellSearch®, Tumor Phenotyping Reagent EGFR	Baseline: median 8 cells/7.5mL (range 0-184). Significant correlation with PSA>30 ng/ml, EOD>3, ISUP 5. For CTC<5, OS 20 mo. For CTC ≥5, OS 11.5 mo.	EGFR+ CTC: median 39% (range 5 – 100%)
D. C. Danila et al., 2016. [16]	Clinical Validity of Detecting Circulating Tumor Cells by AdnaTest Assay Compared With Direct Detection of Tumor mRNA in Stabilized Whole Blood, as a Biomarker Predicting Overall Survival for Metastatic Castration-Resistant Prostate Cancer Patients	2b	47 patients, 55 blood samples/ 12 healthy volunteers	mCRPC. Median OS 13 mo (range 1 – 42)	1) AdnaTest 2) Direct detection PCR 3) CellSearch	3) Unfavourable (≥5 cells/7.5 mL) in 45% (95% CI 33 – 58%). Significant disagreement 1 & 3 (p=0.027), 2 & 3 (p=0.001)	1) Specificity 100%. Positive in 62% (95%CI 48 – 75%) based on KLK3, EGFR, PSMA. 2) Positive in 69% (95%CI 55 – 81%) based on KLK3, KLK2, HOXB13, FOXA1. No disagreement between 1 & 2 (p=0.42).
C. Massard	Phenotypic and genetic heterogeneity of tumor tissue and	4	55 patients	mCRPC, testosterone <0.50 ng/ml, ECOG 0–2	1) CellSearch 2) ISET	1) Median 72 CTC/7.5 mL (range 15 –	1) AR-amplification in 4/6 patients.

et al., 2016. [31]	circulating tumor cells in patients with metastatic castration-resistant prostate cancer: A report from the PETRUS prospective study					249). Epithelial cells 2) Median 45 CTC/7.5 mL (range 4 – 218) with no epithelial or mesenchymal markers	ERG-rearrangement CTC 88% concordant to tumor
T.M. Gorges et al., 2016. [19]	Heterogeneous PSMA expression on circulating tumor cells - a potential basis for stratification and monitoring of PSMA-directed therapies in prostate cancer	2b	29 patients	Metastatic PC, different therapy	1) CellSearch, 2) IF for PSMA	20 patients (69%) CTC-positive.	12 patients with PSMA-positive CTC
D. Lorente et al., 2016. [28]	Decline in Circulating Tumor Cell Count and Treatment Outcome in Advanced Prostate Cancer	2b	486 patients with baseline CTC ≥5 cells/7.5 ml	mCRPC, chemotherapy or placebo. Median follow-up 11.2 mo, median OS 11.6 mo.	CellSearch. CTC measured baseline and at therapy	Median baseline CTC 19.5 cells/7.5 ml. 30% decline associated with better OS (16.1 vs 9.7 mo; HR 0.39, 95% CI 0.3–0.5; $p < 0.001$). No difference between stable and progressive CTC count.	--
N. J. Vogelzang et al., 2017. [46]	Circulating Tumor Cells in a Phase 3 Study of Docetaxel and Prednisone with or without	4	208 patients with available baseline CTC counts	mCRPC, docetaxel + 1)lenalidomide or 2)placebo	CellSearch.	Baseline CTC ≥5 cells/7.5 ml is prognostic for OS	--

	Lenalidomide in Metastatic Castration-resistant Prostate Cancer					(HR 3.23, p = 0.0028).<br><br>Mean response<br>1) -69.7 cells/7.5 l,<br>2) -34.1 cells/7.5 ml	
H. Beltran et al., 2016. [14]	The Initial Detection and Partial Characterization of Circulating Tumor Cells in Neuroendocrine Prostate Cancer	1b	27 patients to develop classification, 159 for validation	Pure or mixed neuroendocrine metastatic PC (NEPC), mCRPC	1) CellSearch and Epic<br>2) IF for CK, CD45, AR, and DAPI	In NEPC+ median CTC 64.4, in NEPC- median CTC 4.2, p<0.01.	Patients with visceral mts: 35% CTC NEPC+, 15% NEPC-, p=0.04
H. I. Scher et al., 2016. [40]	Association of AR-V7 on Circulating Tumor Cells<br>as a Treatment-Specific Biomarker With Outcomes and Survival in Castration-Resistant Prostate Cancer	2b	161 patients, 193 blood samples	Progressive mCRPC undergoing a change in treatment	IF for DNA, CK, CD45, and AR-V7	AR-V7+ median count 2.4/mL (range 0.74 – 105 cells/mL).<br>1st line therapy: AR-V7+ at 3% samples,<br>2nd – at 18%,<br>3rd – at 31%.	0 samples with PSA response AR-V7+.<br>20% samples with resistant PC AR-V7+.<br><br>Worse outcomes for AR-V7+ at ARSI (median PFS 2.3 vs 14.5 mo, p < 0.001; median OS 4.6 mo vs not reached, p < 0.001)<br><br>Similar outcomes for AR-V7+ at taxanes.
H. I. Scher et al., 2017. [39]	Phenotypic Heterogeneity of Circulating Tumor Cells Informs Clinical Decisions between AR Signaling	2b	179 patients, 319 blood samples	Progressive mCRPC undergoing a change in treatment	IF for DNA, CK, CD45, and AR-V7.<br>SeqPlex (Sigma) for single-cell	--	Median OS for "high" and "low" Shannon index:<br>- at ARSI 8.8 mo vs. 28.1 mo, p = 0.0015).

	Inhibitors and Taxanes in Metastatic Prostate Cancer				whole genome amplification (WGA)		- at taxanes, 11.4 mo vs. 12.9 mo, p = 1.
L. León-Mateos et al., 2017. [26]	Improving circulating tumor cells enumeration and characterization to predict outcome in first line chemotherapy mCRPC patients	2b	29 patients, 19 healthy individuals	mCRPC eligible for Docetaxel / Cabazitaxel	1) CellSearch, 2) EpCAM–based inmunoisolation (CELLectionTM) and a RT-qPCR analysis	CTC detected in 93.1% patients, ≥ 5 CTC/mL in 65.5% patients (100% with biochemical and 75% with radiological progression). Positive correlation with T3, N+.	Shorter PFS for high levels of KLK3, AR, CYP19 and GDF15. High GDF15 level increased risk of progression a 2.5-fold and risk of death 15.7-fold.
A. Josefsson et al., 2017. [23]	Circulating Tumor Cells as a Marker for Progression-Free Survival in Metastatic Castration-Naïve Prostate Cancer	2b	53 patients	Castration - naïve with PSA >80 ng/ml and/or metastatic PC	1) AdnaTest Prostate Cancer Select/Detect + PCR for PSA, PSMA, EGFR, and actin 2) CellSearch, enrichment with EpCAM-antibody. IF for CD45 and CK	1) CTC detected in 95% (100% with mts), 2) CTC detected in 80% 1) CTC detection predicted mts with a sensitivity 98%, specificity 75%, PPV 96%, and NPV 86%	100% positive for PSA mRNA expression, 64% - for PSMA, 23% - for EGFR
S. K. Pal et al., 2018. [36]	Synaptophysin expression on circulating tumor cells in patients with castration resistant	4	44 patients	mCRPC	CellSearch + Synaptophysin (Syp) antibody	CTC count significantly higher in Abiraterone or Enzalutamide	SYP-positive CTC percentage higher in resistant, than in response group.

	prostate cancer undergoing treatment with abiraterone acetate or enzalutamide					resistant, than in response group.	
L. Xu et al., 2017. [48]	The novel association of circulating tumor cells and circulating megakaryocytes with prostate cancer prognosis	2b	81 patients, 24 healthy male donors	1) 38 with untreated localized PC, 2) 43 with progressive CRPC (40 with mts)	1) ParsortixTM (isolation) 2) IF for CK, VIM, CD45.	--	1) CK+/VIM- 47%, CK+/VIM+ 18%, CK-/VIM+ 55% 2) CK+/VIM- 72%, CK+/VIM+ 70%, CK-/VIM+ 88%. No association with OS
E. S. Antonarakis et al., 2017. [8]	Clinical Significance of Androgen Receptor Splice Variant-7 RNA Detection in Circulating Tumor Cells of Men With Metastatic Castration-Resistant Prostate Cancer Treated With First- and Second-Line Abiraterone and Enzalutamide	4	202 patients (95 abiraterone, 107 enzalutamide)	mCRPC starting treatment with enzalutamide or abiraterone	AdnaTest, EpCAM –based CTC capture. Detection of AR-V7	73.8% patients CTC+.<br><br>Response in 75.5% CTC-.	17.8% patients CTC+/AR-V7+.<br><br>Response in 52.2% CTC+/AR-V7-, 13.9% CTC+/ARV7+.<br><br>AR-V7 is an independent predictor of PFS and OS.
M. K. Thakur et al., 2018. [44]	Phase I Trial of the Combination of Docetaxel, Prednisone, and Pasireotide in Metastatic Castrate-Resistant Prostate Cancer	4	18 patients (7 previously underwent radical prostatectomy, 8 had neuroendocrine features)	mCRPC, combination of docetaxel, prednisone, and pasireotide	--	Baseline <5 CTC/ 7.5mL – 6 patients, median OS 26.6 mo. Baseline ≥5 CTC/ 7.5mL – 9 patients, median OS 14.6 mo.	--
P. C. Barata et	Phase I/II study evaluating the safety and clinical efficacy of	2b	21 patients total, 11 had CTC	mCRPC progressed after 1 or 2 prior chemotherapy regimens	CellSearch	Baseline median 80 CTC (range 17–397),	--

al., 2019. [12]	temsirolimus and bevacizumab in patients with chemotherapy refractory metastatic castration-resistant prostate cancer		assessment baseline	(docetaxel or mitoxantrone)		no association with Gleason score and PSA.<br><br>Median relative decrease 82% (range 62–100%) at 3 cycle. No correlation with PSA change.	
D. Lorente et al., 2018. [27]	Circulating Tumor Cell Increase as a Biomarker of Disease Progression in Metastatic Castration-Resistant Prostate Cancer Patients with Low Baseline CTC Counts	4	511 patients	mCRPC at chemotherapy or abiraterone, CTC counts < 5 cells/7.5 mL	CellSearch	OS associated with CTC count, HR 1.65; 95% CI 1.32 – 2.05; p<0.001.<br><br>Worse OS for CTC progression in 4 weeks, 8 weeks, and 12 weeks (27.1 vs 13.6 mo; HR 3.9 [95%CI: 2.9-5.2]; p<0.001)	--
S. Tommasi et al., 2019. [45]	Standardization of CTC AR-V7 PCR assay and evaluation of its role in castration resistant prostate cancer progression	1b	44 patients in validation cohort	mCRPC, abiraterone / enzalutamide or chemotherapy	AdnaTest, PCR for PSA and PSMA	--	75% AR-V7+ patients resistant for abiraterone / enzalutamide, 57% AR-V7− patients hormone sensitive. Median PFS 6 mo for AR-V7+ and 18 mo for AR-V7-

T. Okegawa et al., 2018. [35]	AR-V7 in circulating tumor cells cluster as a predictive biomarker of abiraterone acetate and enzalutamide treatment in castration-resistant prostate cancer patients	2b	98 patients (64 at abiraterone, 34 at enzalutamide)	mCRPC, abiraterone / enzalutamide + zolendronic acid	1) on-chip multi-imaging flow cytometry<br>2) PCR for AR-V7	CTC- patients: PSA response in 79.6%, median PFS not reached, median OS 35 mo	CTC+/ AR-V7-: PSA response in 43.5%, median PFS 17 mo, median OS 24 mo.<br><br>CTC+/ AR-V7+: PSA response in 15.4%, median PFS 13 mo, median OS 13.5 mo.
D.T. Miyamoto et al., 2018. [32]	An RNA-Based Digital Circulating Tumor Cell Signature Is Predictive of Drug Response and Early Dissemination in Prostate Cancer	4	27 patients	mCRPC, initiating abiraterone therapy in the first-line setting	1) Digital CTCM score<br>2) CTC-iChip	Elevated CTCM score predicts worse OS (HR = 6.0; P = 0.01) and rapid radiographic progression (HR = 3.2; P = 0.046)	Expression of the genes predicts lower OS: HOXB13 (HR = 11.8; p = 0.004), FOLH1(HR = 6.3; p = 0.01), KLK2 (HR = 6.0; p = 0.01), KLK3 (HR = 8.3; p = 0.02), AGR2 (HR = 5.6; p = 0.01);<br><br>ARV7 >14.7 transcripts/mL in CTCs predicts shorter OS (HR = 28.8; P < 0.001) and radiographic PFS (HR = 10.4; p = 0.004)
H.I. Scher et al., 2018. [38]	Assessment of the Validity of Nuclear-Localized Androgen Receptor Splice Variant 7 in Circulating Tumor Cells as a Predictive	2b	142 patients (70 ARSI; 72 taxanes)	mCRPC before starting ARS inhibitors or taxanes	IHC for DNA, CK, CD45, AR-V7	--	Median OS AR-V7-: 19.8 mo for ARSI vs 12.8 mo for taxane (HR, 1.67; 95% CI, 1.00-2.81; p = 0.05)<br>Median OS AR-V7+: 14.3 mo taxanes vs

	Biomarker for Castration-Resistant Prostate Cancer						7.3 mo ARSI (HR 0.62; 95% CI, 0.28-1.39; p = 0.25)
K.A. Autio et al., 2018. [11]	Safety and Efficacy of BIND-014, a Docetaxel Nanoparticle Targeting Prostate-Specific Membrane Antigen for Patients With Metastatic Castration-Resistant Prostate Cancer: A Phase 2 Clinical Trial.	4	42 chemotherapy-naïve patients with progressing mCRPC after 2-line HT	mCRPC, 20 - previous treatment with abiraterone acetate, 5 - enzalutamide, 6 both abiraterone and enzalutamide, and 12 - Ra 223.	Epic Sciences non-EPCAM-based CTC detection platform	89% patients CTC+<br><br>OS 9.5 mo for CTC count above median vs 28.2 mo for CTC count below median CTC, p < 0.001.	--
B. De Laere et al., 2018. [18]	Circulating tumor cells and survival in abiraterone- and enzalutamide-treated patients with castration-resistant prostate cancer.	2b	102 patients	mCRPC, abiraterone / enzalutamide. Follow-up 10-12 weeks	1)CellSave<br>2) CellPrep system,<br>3)CellTracks<br>4)Analyzer II	For CTC increase vs decreasing or stable CTC count shorter:<br>- PFS, 4.03 vs 12.98 vs 13.67 mo, HR 3.6, 95%CI 1.9-6.8; p < 0.0001)<br>-OS, 11.2 mo vs not reached, HR 9.5, 95%CI 3.7-24; p < 0.0001)	--
A. J. Armstrong et al, 2019. [9]	Prospective Multicenter Validation of Androgen Receptor Splice Variant 7 and Hormone Therapy Resistance in High-Risk Castration-Resistant Prostate	1b	118 patients	MCRPC, abiraterone / enzalutamide / both	1) CellSearch<br>2)AdnaTest mRNA	--	Median OS for AR-V7+ vs AR-V7- : 10.8 vs 27.2 mo (HR, 3.9; 95% CI, 2.2 to 6.9).

	Cancer: The PROPHECY Study.						
B. P. S. Belderbos et al., 2019. [13]	Associations between AR-V7 status in circulating tumour cells, circulating tumour cell count and survival in men with metastatic castration-resistant prostate cancer.	2b	94 patients	mCRPC, patients after docetaxel or cabazitaxel	CellSearch	Median OS for ≥5 CTCs: 6.9 mo (IQR: 4.3-13.8) vs 22.3 mo, IQR: 19.2-34.6).	No difference in survival between AR-V7+ and AR-V7- patients (HR of 1.1, 95% CI: 0.6-1.9, p= 0.78).
P. Sepe et al., 2019. [41]	Could Circulating Tumor Cells and ARV7 Detection Improve Clinical Decisions in Metastatic Castration-Resistant Prostate Cancer? The Istituto Nazionale dei Tumori (INT) Experience.	4	37 patients	mCRPC, enzalutamide/abirateron	1)Enrichment – AdnaTest. 2)Semiquantitative multiplex PCR using the PrimerMix provided in the AdnaTest.	Radiological response: 66% for CTC-, 25% for CTC+.<br><br>OS 14.6 mo for CTC+ vs >24 mo for CTC-.<br><br>HR for relapse 3.85 fold higher	Radiological response similar for ARV7- and ARV7+.<br><br>AR+ HR for reccurence 3-4-fold higher, for death 11-fold higher
S.T. Tagawa et al., 2019. [43]	Expression of AR-V7 and ARv567es in Circulating Tumor Cells Correlates with Outcomes to Taxane Therapy in Men with Metastatic Prostate Cancer Treated in TAXYNERGY.	4	54 chemotherapy-naïve patients with progressive mCRPC.	mCRPC, docetaxel/cabazetaxel	ddPCR(digital PCR)	--	Median PFS 16.6 mo for double-negative patients, 11.2 mo for ARV7-/ARv567es+ (p=0.18), and 8.5 mo for ARV7+ (p=0.004). Median PFS ARV7 vs ARV7+ : 12.0 vs 8.5 mo (HR=0.38, P=0.01). For the ARv567es- vs ARv567es+, median PFS 12.7 vs 7.3 mo

							(HR=0.37, p=0.02).
I.E. de Kruijff et al., 2019. [17]	Circulating Tumor Cell Enumeration and Characterization in Metastatic Castration-Resistant Prostate Cancer Patients Treated with Cabazitaxel.	4	114 patients	mCRPC previously treated with docetaxel. 36% of the patients had received anti-AR treatment (mainly abiraterone 23/43, 53%) for mCRPC before enrollment. 6 weeks of cabazitaxel therapy.	1)CellSave, 2)CellSearch, 3)AllPrep DNA/RNA Micro Kit	Median PFS: group 1 (<5 CTCs at baseline and follow-up (n = 24)): 8.7 mo; group 2 (≥5 CTCs at baseline, <5 CTCs at therapy (n = 19)): 6.4 mo; group 3 (<5 CTCs at baseline, ≥5 CTCs at therapy (n = 5)): 7.4 mo; group 4 (≥ 5 CTCs both at baseline and during therapy (n = 47)): 3.5 mo. Median OS group 1: 19.0 mo; group 2: 12.8 mo; group 3: 23.0 mo; group 4: 6.9 mo.	--
P. C. Mandel et al., 2021. [29]	Enumeration and Changes in Circulating Tumor Cells and Their Prognostic Value in Patients Undergoing	4	33 patients with hormone-naïve oligometastatic PC	mCRPC, cytoreductive RP	CellSearch	CTC-/CTC+ before and after RP was not statistically different (p = 0.285).	--

	Cytoreductive Radical Prostatectomy for Oligometastatic Prostate Cancer-Translational Research Results from the Prospective ProMPT trial.					≥2 CTC associated with worse OS and progression to mCRPC	
A. Sharp et al., 2019. [42]	Clinical Utility of Circulating Tumour Cell Androgen Receptor Splice Variant-7 Status in Metastatic Castration-resistant Prostate Cancer.	2b	136 patients	mCRPC	1)CellSearch 2)AdnaTest	OS in CTC+/AR-V7+ patients compared with CTC- patients (HR 2.13; 95% CI 1.23–3.71; p = 0.02)	OS in CTC+/AR-V7- patients (HR 1.26; 95% CI 0.73-2.17; p=0.4).
R.P. Graf et al., 2020. [20]	Clinical Utility of the Nuclear-localized AR-V7 Biomarker in Circulating Tumor Cells in Improving Physician Treatment Choice in Castration-resistant Prostate Cancer.	2b	193 patients	mCRPC, ARSI/taxanes	Automated immunofluorescent staining for DNA, cytokeratins, CD45, and AR-V7	--	Superior OS for AR-V7+ on a taxane vs ARSI (median 9.8 vs 5.7 mo; HR 1.8; p = 0.041)
J. S. Chung et al., 2019. [15]	Circulating Tumor Cell-Based Molecular Classifier for Predicting Resistance to Abiraterone and Enzalutamide in Metastatic Castration-Resistant Prostate Cancer.	4	37 patients	mCRPC, abiraterone/enzalutamide	1)Anti-EpCAM antibody–conjugated microbeads 2)Multiplex qPCR	In CTC+ median 91 days (IQR: 55-228) to PSA progression and 142 days (IQR: 42-492) to radioclinical progression	the AUCs for the multigene model (AR, AR-V7, PSA, PSCA, TSPAN8, WNT5B, NKX3-1, and SPINK1) showed increased accuracy compared to AR-V7 alone for PSA PFS (0.84 vs. 0.65) and radioclinical PFS (0.86 vs. 0.64).

M. Kozminsky et al., 2019. [24]	Detection of CTC Clusters and a Dedifferentiated RNA-Expression Survival Signature in Prostate Cancer.	4	41 patients	mCRPC	RT-qPCR	--	For OS, the three-gene combination (CDH1, CD11B, and STAT3) showed the best performance (HR: 0.78, 95% CI: 0.62–0.99; HR: 2.04, 95% CI: 1.32–3.14; and HR: 0.46, 95% CI: 0.30–0.72, respectively).
A. Josefsson et al., 2019. [22]	AR-V7 expression in circulating tumor cells as a potential prognostic marker in metastatic hormone-sensitive prostate cancer.	4	36 patients	mCRPC	qRT-PCR	--	Median time to CRPC for AR-V7+ 4.9 mo (95% CI: 3.95 – 9), for AR-V7- 8.9 mo (95% CI: 5.9–12.3) ($p = 0.02$). CSS for AR-V7+ 14.3 mo (95% CI: 7.8–20.7), for AR-V7- 33.0 mo (95% CI: 18.3–47.7) ($p = 0.002$). AR+ ($p = 0.02$, HR: 2.5, 95% CI: 1.2–5.3) and ARV7+ ($p = 0.02$, HR: 2.7, 95%CI: 1.2–6.5) are prognostic.
A.J. Armstrong et al., 2020. [10]	Prospective Multicenter Study of Circulating Tumor Cell AR-V7 and Taxane Versus Hormonal Treatment Outcomes in Metastatic Castration-	2b	118 patients	mCRPC, abiraterone or enzalutamide, 51 - subsequent docetaxel (42) or cabazitaxel (9)	1) Johns Hopkins modified AdnaTest CTC AR-V7 messenger RNA assay 2) the Epic Sciences CTC nuclear-localized	--	Abiraterone or Enzalutamide: AR-V7 + Median OS 11.1/8.4; Median PFS 3.7/3.7; AR-V7 – Median OS 24.8/20.5; Median PFS 7.2/6.0;

	Resistant Prostate Cancer				AR-V7 protein assay		Taxane Chemotherapy: AR-V7 + Median OS 8.2/6.8; Median PFS 4.0/4.5; AR-V7 – Median OS 12.6/11.1; Median PFS 6.1/5.3.
L. Hofmann et al., 2020. [21]	A Multi-Analyte Approach for Improved Sensitivity of Liquid Biopsies in Prostate Cancer	4	19 patients	Progressive-advanced or metastatic CRPC, before and after analysis: abiraterone, enzalutamide, taxane or combinations	1) the CellCollector 2) RT-qPCR-based 3) shallow whole-genome sequencing (sWGS) of plasma DNA.	No correlation between CTC and PSA or OS	None of the identified biomarkers was significantly associated with OS.
L. León-Mateos et al., 2020. [25]	Global Gene Expression Characterization of Circulating Tumor Cells in Metastasic Castration-Resistant Prostate Cancer Patients	4	28 patients	mCRPC; docetaxel	1) CELLectionTM Epithelial Enrich system 2) CellSearch system 3) qRT-PCR	--	Longer PFS at low expression of MAOA (8.73 mo vs 6.47 mo; p=0.028), MOSPD1 (8.74 mo vs 5.39 mo; p=0.028), QKI (8.78 mo vs 5.74 mo; p=0.022), SDK1 (8.89 mo vs 4.7 mo; p=0.014). Shorter OS for high expression of HOXB13 (16.39 mo vs 31.77 mo; p=0.0014)
M. Marín-Aguilera	Androgen Receptor and Its Splicing Variant 7 Expression in Peripheral Blood	4	81 patients	mCRPC, 71 docetaxel/10 cabazitaxel (11both)	1) IsoFlux System 2) qRT-PCR	--	High ARV7 correlated with better PSA-PFS (HR 0.45, 95% CI 0.3 –

et al., 2020. [30]	Mononuclear Cells and in Circulating Tumor Cells in Metastatic Castration-Resistant Prostate Cancer						0.7, p = 0.002), RX-PFS (HR 0.56, 95% CI 0.3–0.9, p = 0.02) and OS (HR 0.54, 95% CI 0.3–0.9, p = 0.013). High ARV7 expression correlated with worse PFS (p = 0.002) High ARFL correlated with worse OS (p = 0.012).
N. Nagaya et al., 2020. [33]	Prostate-specific membrane antigen in circulating tumor cells is a new poor prognostic marker for castration-resistant prostate cancer.	4	56 patients	mCRPC	AdnaTest	--	For PSMA+ Median PSA-PFS shorter (12 weeks vs 30 weeks, p = 0.008). Median OS shorter (13 mo vs 27 mo, p = 0.01). Shorter PSA-PFS (HR 4.02; 95% CI, 1.33 to 12.8; p = 0.014) Shorter OS (HR 7.62; 95% CI, 1.08 to 153; p = 0.04).
C. Wang et al., 2021. [47]	Improved Prognostic Stratification Using Circulating Tumor Cell Clusters in Patients with Metastatic Castration-Resistant Prostate Cancer	4	64 patients	mCRPC, androgen receptor signaling inhibitors or chemotherapy	CellSearch	For ≥5 CTC/7.5 mL: Lower PFS (HR 4.03, 95% CI 2.09 – 7.77, p < 0.0001), Lower OS (HR 32.84, 95% CI	--

						8.42–128.2, p < 0.0001). Higher risk for death (HR 6.9, 95% CI 2.72–17.47).	
T. Pereira-Veiga et al., 2021. [37]	Longitudinal CTCs gene expression analysis on metastatic castration-resistant prostate cancer patients treated with docetaxel reveals new potential prognosis markers.	4	20 patients	mCRPC, docetaxel	CellSearch, AllPrep DNA/RNA Mini Kit - Extraction of RNA, TaqMan Gene Expression Master Mix – cDNA expression	Patients with ≥ 5 CTCs tended to a shorter OS and PFS.	Worse PFS for high expression of KRT19 (139 vs 180 days, p=0.03), ZEB1 (119 vs 190 days, p=0.03), CDK4. Lower OS for ZEB1 (p=0.04, 260 vs 426 days), KRT19 or KLK3 (74 vs 391 days, p=0.008 and 183 vs 720 days, p=0.02).
W. Chong et al., 2021. [49]	Integration of circulating tumor cell and neutrophil-lymphocyte ratio to identify high-risk metastatic castration-resistant prostate cancer patients	4	63 patients	mCRPC	CellSearch. IHS for DAPI, CD45, CK-8, 18, 19.	CTC+ (58.7% patients) had lower PFS (5 mo vs. 18.1 mo., p < 0.001) 4.02-fold risk of progression (HR 4.02, 95% CI 2.05 to 7.86) lower OS (14.2 mo. vs. not reached, p = 0.006)	--
G. Francolini et al., 2021 [51]	Prospective assessment of AR splice variant and PSMA detection on circulating tumor cells	2b	28 patients	mCRPC on standard treatment (ARTA + ADT) after I line ADT failure	AdnaTest AR-V7	Median time to castrate resistance shorted for CTC+, 32.3 vs	Time for castrate resistance: No difference for AR-V7+ and PSMA+ CTC

	of mCRPC patients: preliminary analysis of patients enrolled in PRIMERA trial (NCT04188275)					75 mo., p = 0.03).	Shorter for ARFL+, 30.2 vs 51.1 mo., p = 0.02.
B. Hayes et al., 2021 [50]	Circulating Tumour Cell Numbers Correlate with Platelet Count and Circulating Lymphocyte Subsets in Men with Advanced Prostate Cancer: Data from the ExPeCT Clinical Trial (CTRIAL-IE 15-21)	2b	61 patients	mPCa with no history of RP	ScreenCell, flow cytometry	CTC number correlates with lymphocytes count (r2 = 0.17) and NK count (r2 = 0.5).	--
M. Ladurner et al., 2021 [52]	Validation of Cell-Free RNA and Circulating Tumor Cells for Molecular Marker Analysis in Metastatic Prostate Cancer	2b	41 patients, 56 blood samples	mPCa	ScreenCell or Parsortix™	CTC count higher in mCRPC (mean CTCs 16.4 ± 4.9) vs mHSPC (mean CTCs 5.7 ± 1.1). No correlation with PSA, Gleason	--
G. Di Lorenzo et al., 2021 [53]	Assessment of Total, PTEN –, and AR-V7+ Circulating Tumor Cell Count by Flow Cytometry in Patients with Metastatic Castration-Resistant Prostate Cancer Receiving Enzalutamide	2b	45 patients	mCRPC scheduled to start enzalutamide	Anti-Cytokeratins 8/18, PTEN, and AR-V7 antibodies. Western blot, Flow Cytometry	84.4% CTC+ For >5 CTC worse rPFS (HR, 2.35; 95% CI, 1.14-4.84; p = 0.021) and OS (HR, 3.08; 95% CI, 1.45-6.54; p = 0.003)	71.1% PTEN–, 51.1% AR-V7 + For ≥2 PTEN– worse rPFS (HR, 3.96; 95% CI, 1.8-8.72; p = 0.001) and OS (HR, 2.36; 95% CI, 1.12-5; p = 0.025) For ≥1 AR-V7+ worse rPFS (HR, 5.05; 95% CI, 2.4-10.64; P < .001)

D. Maillet et al., 2021 [54]	Her2 Expression in Circulating Tumor Cells Is Associated with Poor Outcomes in Patients with Metastatic Castration-Resistant Prostate Cancer	4	41 patients	mCRPC patients (including 31 treated with Androgen Receptor Signaling Inhibitors [ARSI])	AdnaTest	88% CTC+. For CTC+: Shorter median PSA-PFS, 6.2 mo. vs 23 mo., HR = 3; 95% CI 1.2–7.4; p = 0.016. Shorter OS, median 21.9 mo vs not reached, p = 0.016.	56% AR-V7+, 63% Her2+. For Her2+: shorter median PSA-PFS, 6.2 mo vs 13 mo., p = 0.034. shorter OS, median 22.7 mo. vs not reached, p = 0.05.

Table S2. CTC in non-metastatic PC.

Author, year	Title	LE	Number of patients/samples	Conditions	Methodology	Cell count	Genes/receptors
J. W. Davis et al., 2008. [55]	Circulating Tumor Cells in Peripheral Blood Samples From Patients With Increased Serum Prostate Specific Antigen: Initial Results in Early Prostate Cancer	2b	1) 97 patients, 2) 25 control	Localized PCa before RP, control - elevated PSA without PCa	CellSearch before and after surgery	Same CTC count for group 1 and 2. CTC+ 21% and 20%, p= 0.95. No difference before and after surgery (16% vs 10%, p= 0.51)	--
C. P. Meyer et al., 2016. [57]	Limited prognostic value of preoperative circulating tumor cells for early biochemical recurrence in patients with localized prostate cancer	4	152 patients	PC without HT	CellSearch	CTC detected in 11.2% patients. No significant correlation with PSA, stage, BCR.	--
A. Kuske et al., 2016. [56]	Improved detection of circulating tumor cells in non-metastatic high-risk prostate cancer patient	2b	86 patients before RP, 52 patients 3 mo after RP	High-risk PC (D'Amico criteria)	1)CellSearch 2)CellCollector 3)EPISPOT assay	CTC detection: 1)37% (51/138, range 1–10, median 1.8); 2)54.9% (62/113, range 1–12, median 2.4); 3)58.7% (74/126, range 1–13, median 3). The concordance between all three methods 37.4%, paired concordance 56–60%. 3) CTC correlated with PSA, stage.	--

						No correlation for 1) and 2)	
T. Todenhöfer et al., 2016. [58]	Microfluidic enrichment of circulating tumor cells in patients with clinically localized prostate cancer	4	50 consecutive patients	Patients before RP	Enrichment with microfluidic ratchet device. Staining for CK, EpCAM, CD45, DAPI.	50% CTC+. No correlation of CTC presence or concentration with PSA, stage (T or N), Gleason.	All cells EpCAM+
G. Roviello et al., 2017. [59]	Circulating tumor cells correlate with patterns of recurrence in patients with hormone-sensitive prostate cancer	2b	42 patients	Advanced PC with recurrence after RP of radiotherapy	CellSearch	33.3% CTC+. Correlation with bone mts, OR = 4.0; 95% CI 1.0–15.9, p=0.05). No correlation with biochemical reccurence.	--
H. Tsumura et al., 2017. [60]	Perioperative Search for Circulating Tumor Cells in Patients Undergoing Prostate Brachytherapy for Clinically Nonmetastatic Prostate Cancer	4	59 patients	Patients undergoing high-dose or low-dose brachytherapy, cM_0	CellSearch	Before surgery – 0. During surgery 11.8% patients CTC+ (p=0.012). No correlation with any clinicopathological and perioperative variables	--
N. P. Murray et al., 2018. [62]	Minimum Residual Disease in Patients Post Radical Prostatectomy for Prostate Cancer: Theoretical Considerations, Clinical Implications and Treatment Outcome	2b	321 patients	1 mo after RP.	Differential centrifugation with Histopaque. IHC for PSA	39.6% patients CTC+. OS 1) CTC- and micro mts- 92.7% (86.3 – 96.2%) 2) CTC- and micro mts+ 55.8% (37.2 – 70.9%) 3) CTC+ and micro mts- 6.41% (1.19 – 18.21%)	--

						4) CTC+ and micro mts+ 3.57% (3.52 – 3.63%)	
N. P. Murray et al., 2018. [61]	10 Year Biochemical Failure Free Survival of Men with CD82 Positive Primary Circulating Prostate Cells Treated by Radical Prostatectomy	4	285 patients	RP as a sole treatment	Differential centrifugation with Histopaque. IHC for PSA and CD82	78.6% patients CTC+. 36, 60 and 120 mo survival for CTC- 98%, 96%, 90%.	20% patients CD82+. 36, 60 and 120 mo survival for CTC+ CD82- 93%, 93%, 69%. For CTC+ CD82+ 62%, 44%, 16%
S. Y. Choi et al., 2019. [63]	Circulating Tumor Cell Counts in Patients With Localized Prostate Cancer Including Those Under Active Surveillance	2b	45 patients, 17 healthy volunteers	RP	1) CTC isolation kit and SMART BIOPSY™ Cell Isolator 2) IHS for PSMA-EpCAM+ and CD45	--	EpCAM+ CTC: 40% sensitivity, 88.2% specificity, 53.2% accuracy, 90% PPV, and 35.7% NPV. PSMA+ CTC: 2.2% sensitivity, 100% specificity, 29% accuracy, 100% PPV, and 27.9% NPV. Positive correlation of grade and EpCAM
S. S. Salami et al., 2019. [64]	Circulating Tumor Cells as a Predictor of Treatment Response in Clinically Localized Prostate Cancer	2b	49 patients	Radiotherapy + HT (42%) or RP (58%)	1) Epic Sciences CTC platform. 2) IHS for CK, CD45, AR. 3) Sigma SeqPlex enhanced whole-genome amplification kit	Significantly more CTC count in patients with mts	Significantly more AR+ CTC in patients with mts and biochemical relapse
H. Liu et al., 2020. [67]	Prospective Study of the Clinical Impact of Epithelial and	4	80 patients	Predominantly low and	1)CanPatrol™ 2)RNA-in situ hybridization with	55% patients CTC+	38.8% patients with epithelial CTC, 12.5% with

	Mesenchymal Circulating Tumor Cells in Localized Prostate Cancer			intermediate risk	epithelial markers (EpCAM, CK 8/18/19), mezenchymal markers (Vimentin, Twist), and CD45.		mesenchymal CTC, 47.5% bi-phenotypical CTC.<br><br>Bi-phenotypical CTC count predicted extraprostatic extension, $p<0.01$, OR=1.383, AUC 0.72.
W. A. Cie´slikowski et al., 2020. [65]	Circulating Tumor Cells as a Marker of Disseminated Disease in Patients with Newly Diagnosed High-Risk Prostate Cancer	2c	104 patients	High-risk PC (D'Amico criteria), primary RP or radiotherapy.	1) CellSearch<br>2) Dual fluoro-EPISPOT $^{PSA/FGF2}$<br>3) CellCollector® CANCER01	CTC detection rate:<br>1) 23.9%,<br>2) 52%,<br>3) 57.7%<br>≥4 CTC predicts mts	--
A. Zapatero et al., 2020. [66]	Detection and dynamics of circulating tumor cells in patients with high-risk prostate cancer treated with radiotherapy and hormones: a prospective phase II study	4	65 patients	Treatment-naïve patients without mts, scheduled for radiotherapy + HT	CellSearch	Median CTC count 1 (1–136) cell/7.5 mL.<br><br>CTC detected in 7.5% patients.<br>After HT – 4.6%.<br>After radiotherapy – 0%.<br><br>No association of CTC with OS or other features.	--
S. Knipper et al., 2021 [68]	Possible Role of Circulating Tumour Cells for Prediction of Salvage Lymph Node Dissection Outcome in Patients with Early	4	20 patients	PSMA-radioguided surgery for relapse in pelvic lymph nodes	CellSearch	CTC+:<br>more positive lymph nodes, median<br>8 vs 2.	--

	Prostate Cancer Recurrence					Shorter BFS, 1.4 mo vs 4.3 mo, p = 0.018.	
S. Lian et al, 2021 [69]	Folate-Receptor Positive Circulating umor Cell Is a Potential Diagnostic Marker of Prostate Cancer	2b	30 patients + 7 with bladder cancer and 7 with benign conditions (control)	Treatment-naive sporadic PCa	CytoploRare Kit	--	FR+ CTC in malignant vs benign (12.62 ± 1.2 vs. 6.34 ± 0.64, p < 0.001). AUC 0.864

Table S3. CTC in PC screening and diagnostics.

Author, year	Title	No., of patients/samples	Conditions	Methodology	Cell count	Sensitivity	Specificity
I. Puche-Sanz et al., 2017. [72]	A comprehensive study of circulating tumour cells at the moment of prostate cancer diagnosis: biological and clinical implications of EGFR, AR and SNPs	86 patients	Clinical suspicion of PC, based on PSA screening, and meeting criteria for prostate biopsy (PSA>10 ng/ml or PSA between 4 and 10 ng/ml with a free/total PSA <0.2)	1) CTCs and genetic analysis. (immunohistochemistry, immunofluorescence) 2) A systematic 20-core TRUS-guided biopsy	CTC-positive if ≥1 $CTCs^{CK+}$/10 ml Younger age associated with the CTC+ (p=0.03)	14.2% PPV 31.2%	78.4% NPV 57.4%
S. C. Bhakdi, 2019. [71]	Accuracy of Tumour-Associated Circulating Endothelial Cells as a Screening Biomarker for Clinically Significant Prostate Cancer	146 biopsy-naïve patient, 69 men had PSA in grey zone below 10 ng/mL	Tumour-associated circulating endothelial cells (tCECs) in cases suspicious for PCa; primary definition of csPCa (Gleason 3 + 4) and secondary definition (Gleason 4 + 3)	1) high-gradient magnetic cell separation (hMX) and cryo-immunostaining for tCECs identification 2) A systematic 10-12 core TRUS-guided biopsy	All cases with cell of immunopathological class III (suspicious for malignancy) or higher were considered positive for malignancy	For PSA 4 - 10 and primary definition of csPCa 75% (95% CI 43–95), PPV 32% (16–52); for secondary definition of csPCa 71% (29–96), PPV 18% (6–37)	For PSA 4 – 10 and primary definition of csPCa: 67% (95% CI 53–79), NPV 93% (80-98), 63% (50–75) NPV 95% (83–99)
N. P. Murray et al., 2020. [70]	Platelet-to-lymphocyte ratio and systemic immune-inflammation index versus circulating prostate cells to	1223 patients	114/467 (24.4%) complied with the Epstein criteria for active surveillance. 296 (63.2%) ISUP 1, 145 (31%) were	1) Histopaque 1,077 (Sigma-Al-drich): mononuclear cells (circulating prostate cells-CPC) identification.	CPC positive when 1 CPC/8 ml of blood	0.97 (95%CI 0.94 – 0.98) PPV 65.0%	0.79 (95%CI 0.76 – 0.81) NPV 98.0%

predict significant prostate cancer at first biopsy.	ISUP 2 &3, 27 (5.8%) ISUP 4 &5	2) The 1999 ISHAGE for the CTC's expression of P504S.

Table S4. CTC detection: manufacturers and technology.

Diagnostic system	Manufacturer	FDA or EMEA - approved	Blood sample volume	Blood collection	Enrichment	Characterization & criteria for positivity	Results	Used in
AdnaTest	QIAGEN, Hilgen, Germany.	--	10 mL	EDTA tube placed on ice	Magnetic beads conjugated with EpCAM, HER2 antibodies	KLK3, PSMA, EGFR expression	At least 1 tumor-associated transcript >0.1 ng/ml	[15], [26], [8], [9], [35], [36], [39], [47], [51], [54]
CanPatrol	SurExam, Guangzhou, China	--	5 mL	EDTA tube	Filtration through calibrated membrane with 8-μm diameter pores	Cytomorphology (cell phenotype). Differentiation of epithelial and mesenchymal cells	Absolute number	[59]
CellSearch	Menarini Silicon Biosystems, Huntington Valley, USA	2007	7.5 mL	CellSave tube	Anti-EpCAM coated magnetic nanoparicles	IFM: positive if pan-keratin+ & CD45-	Favorable <5, unfavorable 5 cells / 7.5 mL	[9], [11], [14], [15], [18]–[22], [24], [27]–[30], [36], [40], [42], [44], [45], [47], [48], [57], [58], [60], [62], [63], [85], [49]
CellCollector	Gilupi GmbH, Postdam, Germany	--	N/a (in vivo isolation)	Intravenous canule (30min)	Anti-EpCAM adsorbed onto a metallic wire	IFM: positive if pan-keratin+ & CD45-, eventually PSA+	Absolute number	[56], [13], [57]
CELLection Epithelial Enrich system	Invitrogen (Thermo Fisher Scientific), Waltham, USA	--	1 – 40 mL	CELLlection buffer	Beads coated with EpCAM antibodies	Positive isolation with Biotin Binder Dynabeads	Absolute number	[17]
ELISPOT	Multiple manufacturers, test based on ELISA	--	13-15 mL	EDTA tube	Immuno-density cell isolation (RosetteSepTM STEMCELL Technologies)	protein fingerprinting: positive if PSA or FGF2 secreting cells on primary culture	Absolute number	[56]

EPIC	EpicSciences, San Diego, USA	--	7.5 mL	Streck Tube	None, test on 3 millions nucleated cells	IF: positive if pan-cytokeratine+ & CD45-<br><br>Also tested for AR-V7 splice variant	AR-V7+ if at least one AR-V7+ CTC per two slides assayed	[31], [45], [67], [32], [34], [20]
ISET	Rarecells Diagnostics SAS, Paris, France	--	10 mL	Rarecells® buffer	Antibody-independent isolation by size	Cytomorphology (cell phenotype)	The fixed cells on the ISET© membrane (10 spots). At least 1 cell/10 mL	[82]
IsoFlux	Fluxion Biosciences Inc., Alameda, USA	--	10 mL	EDTA tube	Microfluidic cartridges with magnetic beads conjugated with anti-EpCAM antibodies	IFM: positive if pan-keratin+, nucleus+ & CD45-. Test for EpCAM, EGFR, Mesenchymal, AR-V7	Absolute number	[23]
Parsortix	ANGLE Biosciences, Toronto, Canada	--	10 mL	EDTA tube	Parsortix filtration cassette	Filtration based on size and compressibility. Optional staining.	Absolute number	[43],[52]
ScreenCell	ScreenCell, Sarcelles, France	--	12 mL	EDTA tube	ScreenCell® Cyto filtration	CTCs defined as cells in the same plane of focus as filter pores with nucleus at least twice the diameter of a filter pore	Absolute number	[50],[52]

SMART BIOPSY Cell Isolator	Cytogen Inc., Seoul, South Korea	--	15 mL	Acid citrate dextrose solution	HDM (High density microporous) chip	Size-based filtration	Absolute number	[66]
